# Higher Ustekinumab Levels in Maintenance Therapy are Associated with Greater Mucosal Healing and Mucosal Response in Crohn’s Disease: An Experience of 2 IBD Centers

**DOI:** 10.1093/ibd/izad073

**Published:** 2023-05-09

**Authors:** Ciarán McDonald, Hilary Kerr, Eimear Gibbons, Tincymol Lukose, Danny Cheriyan, Gavin Harewood, Stephen Patchett, Aoibhlinn O’Toole, Orlaith Kelly, Karen Boland

**Affiliations:** Department of Gastroenterology, Beaumont Hospital, RCSI Hospital Group, Dublin 9, Ireland; Department of Gastroenterology, James Connolly Hospital, RCSI Hospital Group, Dublin 15, Ireland; Department of Gastroenterology, James Connolly Hospital, RCSI Hospital Group, Dublin 15, Ireland; Department of Gastroenterology, Beaumont Hospital, RCSI Hospital Group, Dublin 9, Ireland; Department of Gastroenterology, Beaumont Hospital, RCSI Hospital Group, Dublin 9, Ireland; Department of Gastroenterology, Beaumont Hospital, RCSI Hospital Group, Dublin 9, Ireland; Department of Gastroenterology, Beaumont Hospital, RCSI Hospital Group, Dublin 9, Ireland; Department of Gastroenterology, Beaumont Hospital, RCSI Hospital Group, Dublin 9, Ireland; Department of Gastroenterology, James Connolly Hospital, RCSI Hospital Group, Dublin 15, Ireland; Department of Gastroenterology, Beaumont Hospital, RCSI Hospital Group, Dublin 9, Ireland

**Keywords:** ustekinumab, therapeutic drug monitoring, inflammatory bowel disease, mucosal healing, mucosal response, Crohn’s disease

## Abstract

**Background:**

Ustekinumab (UST), a human monoclonal antibody that binds the p40 subunit of interleukin 12 (IL-12) and IL-23, is licensed for induction and maintenance therapy of moderate to severe inflammatory bowel disease (IBD). To date, there is limited data published on any potential association between ustekinumab serum trough levels and mucosal healing in order to guide treatment strategies and appropriate dosing.

**Aim:**

This study aims to identify a relationship between maintenance ustekinumab serum trough levels and mucosal healing and/or response in patients with Crohn’s disease in an observational cohort study.

**Methods:**

Ustekinumab serum trough levels and antibody titres were analyzed in patients on maintenance drug using an ELISA drug-tolerant assay. Mucosal response (MR) was defined as ≥50% reduction in fecal calprotectin level (FC) and/or ≥50% reduction in the Simple Endoscopic Score for Crohn’s Disease (SES-CD score). Mucosal healing (MH) was defined as FC ≤150 µg/mL and/or global SES-CD score ≤5. Median trough levels were analyzed using the Kruskal-Wallis test, and logistic regression was used to determine sensitivity and specificity of levels predicting mucosal response.

**Results:**

Forty-seven patients on maintenance ustekinumab for Crohn’s disease were included in this study. The majority were female (66%), with a median age of 40 years (21-78 years). The majority of patients were biologic-experienced (89.4%, *n* = 42). Patients with histologically confirmed Crohn’s disease represented 100% (*n* = 47) of the cohort. Over one-third of patients (*n* = 18, 38.3%) were on higher than standard dosing of 90 mg every 8 weeks. Patients with mucosal healing (*n *= 30) had significantly higher mean serum ustekinumab levels (5.7 µg/mL, SD 6.4) compared with those with no response (1.1 µg/mL, SD 0.52; *n* = 7, *P* < .0001). A serum ustekinumab trough level greater than 2.3 µg/mL was associated with MH, with a sensitivity of 100% and specificity of 90.6% (likelihood ratio 10.7). Similarly, for patients with MR (*n* = 40), we observed a higher mean serum ustekinumab trough level (5.1 µg/mL, SD 6.1) compared with those with no response (1.1 µg/mL, SD 0.52; *n* = 7, *P* < .0001). Furthermore, a serum ustekinumab trough level greater than 2.3 µg/mL was associated with a 10-fold increased likelihood of mucosal response vs mucosal nonresponse (sensitivity 100%, specificity 90.5%, likelihood ratio 10.5).

**Conclusion:**

This study demonstrates that higher ustekinumab serum trough levels are associated with a greater likelihood of achieving mucosal healing and mucosal response in patients with Crohn’s disease regardless of prior biologic exposure. Further prospective studies are required to correlate target maintenance trough levels and the optimal time to dose-escalate in order to improve patient outcomes.

**Key Messages**

**What is already known?**

Ustekinumab (UST) is licensed for the treatment of mild to moderate inflammatory bowel disease (IBD). However, there is little published data to date regarding ustekinumab serum trough levels and their association with mucosal healing to guide treatment dosing for patients.

**What is new here?**

This observational study demonstrates that higher ustekinumab serum trough levels (above 2.3 μg/mL) are associated with over 10-fold greater likelihood of achieving mucosal healing and mucosal response in patients with Crohn’s disease regardless of prior biologic exposure.

**How can this study help patient care?**

This study contributes to patient care by adding to the literature on therapeutic drug monitoring which can inform bespoke treatment strategies of ustekinumab for patients in order to achieve improved rates of mucosal healing.

## Introduction

Patients with moderate to severe Crohn’s disease (CD) often require biologic therapy for induction and maintenance of remission. Antitumor necrosis factor alpha (anti-TNF-α) agents have been the mainstay of biologic therapy for the past 25 years^1^ and are associated with significantly improved disease-related outcomes and quality of life.^2,3^ Anti-TNF therapy is not without risk, with well documented potential adverse outcomes that need to be considered upon commencement of treatment.^4-9^ Furthermore, approximately 20% to 50% of patients will experience loss of response to anti-TNF therapy at 12 months.^10-12^ An additional 60% of this primary cohort who fail initial anti-TNF therapy will achieve long-term remission on second line anti-TNF therapy.^13,14^ The need for alternative therapeutic pathways and biologic classes in the management of refractory Crohn’s disease has led to approval of alternative therapeutic targets. Ustekinumab is a human monoclonal antibody with a favorable safety profile^15,16^ that binds the p40 subunit of interleukin (IL)-12 and IL-23, thus neutralizing their pro-inflammatory effects on the gastrointestinal mucosa.^17^ Ustekinumab has become established as a viable treatment option for patients with moderate to severe CD following the landmark UNITI-1, UNITI-2, and IM-UNITI studies,^18^ along with patients with moderate to severe UC as demonstrated in the UNIFI^19^ trial.

With the advent of the STRIDE-I^20^ guidance in 2015 and the implementation of “treat to target” for all biologic and biosimilar drugs, therapeutic drug monitoring (TDM) has played an ever increasingly important role in the IBD armamentarium. Maintaining higher trough concentrations of anti-TNF biologics in the absence of anti-drug antibodies is associated with achieving clinical and endoscopic response and a greater likelihood of achieving clinical remission.^21,22^ Similar data regarding potential benefits of drug-level monitoring of ustekinumab in the context of Crohn’s disease are lacking for patients who have had an initial response. This is of particular interest in regions and countries where access to escalated or higher than standard dosing is restricted for financial reasons, and clinicians are asked to provide additional data to support higher doses of ustekinumab or dose escalation in patients achieving response but not remission.

Our aims are to determine whether a threshold maintenance serum concentration for patients with Crohn’s disease is associated with mucosal healing, and whether higher drug levels are associated with increased likelihood of mucosal response or healing.

## Methods

### Study Design and Recruitment

This was a dual-center retrospective observational study of patients on maintenance dose ustekinumab for management of CD between 2016 and 2021. The study was approved by the local research ethics board at each site. Patients were identified from the cohort of confirmed patients with CD at Beaumont Hospital and Connolly Hospital Blanchardstown, which both operate as tertiary IBD centers in Ireland. Patients included were 18 years and older with a histologically confirmed diagnosis of Crohn’s disease and who were on maintenance ustekinumab, defined as having received induction dose (subcutaneous or intravenous) and at least 2 further subcutaneous (maintenance) doses of ustekinumab for a combined minimum treatment duration of 24 weeks. Patients with ulcerative colitis (UC) and inflammatory bowel disease unspecified (IBD-U) were excluded, as were patients with insufficient monitoring data for fecal calprotectin level (FC), endoscopy, and ustekinumab serum trough levels.

### Data Collection

Patients were identified from prospectively maintained treatment databases. A comprehensive chart review was carried out at both sites. Patient demographics were recorded including age, gender, family history of IBD, smoking status, disease phenotype, and age of diagnosis. In addition, Montreal-classified^23^ disease distribution, medication history was noted, and disease activity at the time of ustekinumab induction was recorded using both the endoscopic assessment score via the Simplified Endoscopic Scoring for Crohn’s Disease (SESCD) and fecal calprotectin level. Similar indices of disease monitoring approximate to time of ustekinumab trough level analysis were repeated. Mucosal response (MR) was defined as ≥50% reduction in FC and/or ≥50% reduction in global SES-CD score. Mucosal healing (MH) was defined as FC ≤150 µg/mL and/or global SES-CD score ≤5.

### Ustekinumab Serum Through Level Assays

Patients on maintenance dose subcutaneous ustekinumab having received a minimum of 3 doses (inclusive of induction dose) for management of active luminal or perianal IBD were included in this study. The minimum treatment period evaluated was accordingly 24 weeks. Serum trough levels were drawn during maintenance therapy during a 48-hour window before the patient’s next ustekinumab dose. A drug-tolerant ELISA assay was employed to record trough concentrations and antibody titres.

### Statistical Analysis

Quantitative data were analyzed using median and standard deviation (SD) values with 95% confidence intervals. The Kruskal-Wallis test was used to determine differences between responders and nonresponders to ustekinumab. Using GraphPad Prism, logistic regression was used to determine sensitivity and specificity of levels predicting mucosal response. Receiver operating characteristic (ROC) curve analysis was used to calculate area under the curve (AUC) as a means of assessing performance of serum ustekinumab concentration predicting mucosal healing. A *P* value of < .05 was deemed to be statistically significant.

## Results

### Demographics of Cohort

We identified a total of 72 patients on maintenance ustekinumab across both centers, with 47 patients included in the final analysis after applying selection criteria. Patients with insufficient data measuring disease activity (*n* = 19) were excluded, as were patients with ulcerative colitis (*n* = 4) or inflammatory bowel disease unspecified (*n* = 2). The median age of the cohort was 40 years (21-78 years). The majority were female (66%) and had histologically confirmed CD (100%, *n* = 47). This was a predominantly treatment-refractory cohort with complex disease, as evidenced by the fact that 89.4% were anti-TNF-experienced; and 79% of patients had a stricturing or penetrating disease phenotype (Table 1). Ustekinumab serum trough levels were obtained after a minimum of 3 doses, and median time from blood draw to disease assessment with either FC or endoscopy was 1 month (range 0-13 months). Standardization of time to endoscopy from serum assay was limited by access to endoscopy in the context of the coronavirus disease 2019 (COVID-19) pandemic. The median time from induction of ustekinumab to trough levels was 14 months (range 5-44 months).

**Table 1.** Patient and disease demographics of patient cohort on maintenance ustekinumab.

Variable	Total No. *n* = 47 (% of cohort)
Histological diagnosis: Crohn’s disease	*n* = 47 (100%)
**Gender**	
Female	*n* = 31 (66%)
Median Age	40 yrs (21-78)
**Smoking Status**	
Current	*n* = 4 (8.5%)
Ex-smoker	*n* = 9 (19.1%)
Nonsmoker	*n* = 34 (72.3%)
Extraintestinal manifestations (EIMs)	n = 10 (21.3%)
Anti-TNF experienced	*n* = 42 (89.4%)
Concurrent immunomodulator	*n* = 9 (19.1%)
**Frequencies of previously prescribed biologics**	
IFX	*n* = 33
ADA	*n* = 24
VED	*n* = 4
GOL	*n* = 1
CIC	*n* = 1
TOF	*n* = 1

Abbreviations: IFX, infliximab; ADA, adalimumab; GOL, golimumab; VED, vedolizumab; TOF, tofacitinib; CIC, ciclosporin; UST, ustekinumab

In our patient group, 63.8% (*n* = 30 of 47) achieved mucosal healing, with a further 10 patients (85.1%, *n* = 40 of 47) achieving at least MR during maintenance therapy with ustekinumab. These response rates are striking considering that a high proportion of the overall cohort (*n* = 42 of 47) had previous primary or secondary loss of response to at least 1 anti-TNF therapy along with some failing further biologic agents (Table 1). When biologic-naïve patients are excluded (*n* = 5), this represents an overall mucosal response rate in the anti-TNF-experienced group of 92.9% (*n* = 39 of 42). This proportion reflects patients who remained on ustekinumab after induction, with a minimum of 3 doses received.

Thirty-nine patients received standard first intravenous dose (6 mg/kg) as outlined in the UNITI trial,^18^ and another 8 patients received this as a subcutaneous dose due to regional restrictions at the time in funding of intravenous ustekinumab. Maintenance ustekinumab doses were variable, reflecting the study status as an observational retrospective review of physician practice preferences.

Most patients (59.6%) were maintained on 90 mg of every 8 weeks. Over one-third of patients (*n* = 18, 38.3%) were on higher than standard dosing of 90 mg every 8 weeks. Some patients were dose-escalated to 180 mg of ustekinumab every 8 weeks (*n* = 1) or up to the highest prescribed dose in the patient cohort of 180 mg every 4 weeks (*n* = 9), due to disease burden and physician preference. Seventy percent (*n* = 7 of 10) of patients on higher dosing of 180 mg 8 and 4 weekly had achieved MH, with 100% demonstrating MR. In addition, the mean serum trough level in these 10 patients was higher (16.4 µg/mL) than patients on standard dosing of 90 mg 8-weekly (3.5 µg/mL). Similar dose-related effects on rate of MH were not seen when patients on 90 mg of ustekinumab 90 mg 8 weekly were compared with those on 90 mg 4 weekly (*P* > .05). One patient was prescribed dual biologic therapy with ustekinumab and infliximab, whereas 19.1% (*n* = 9) of patients were treated with concurrent immunomodulator therapy using azathioprine (*n* = 4), 6-mercaptopurine (*n* = 3), and methotrexate (*n* = 2). These immunomodulators combined with ustekinumab did not influence ustekinumab serum trough levels and had no significant impact on likelihood of achieving mucosal healing (*P* = .4). Furthermore, no antidrug antibodies were detected in patient samples included despite use of a drug tolerant assay.

### In Crohn’s Disease, Maintenance Trough Ustekinumab Levels >2.3 µg/mL Were Associated with Mucosal Healing

**Figure 1.**
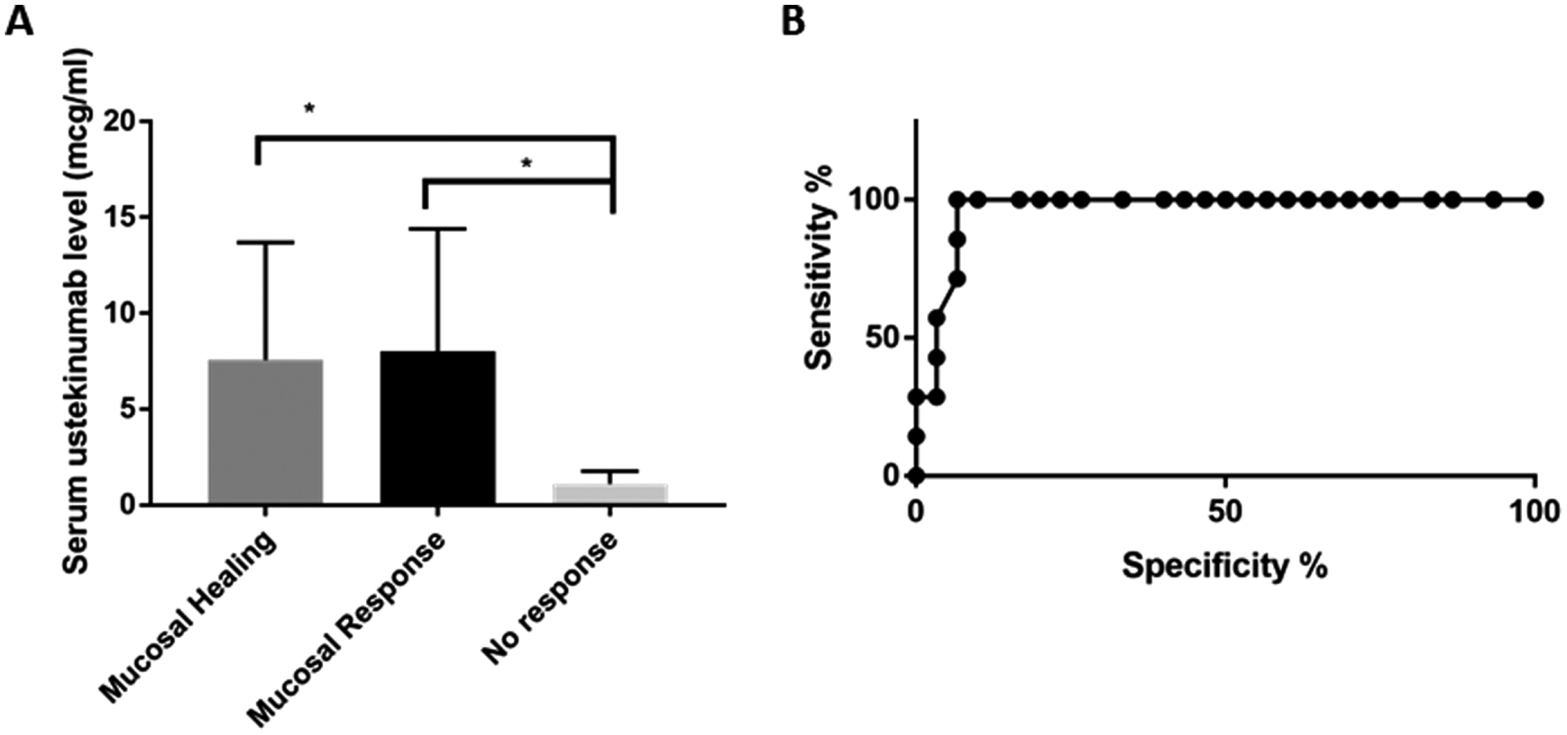
Relationship between serum through levels and mucosal healing or response in patients in Chron’s disease. A, Mean through serum ustekinumab levels with standard deviation in patients who had achieved mucosal healing (*n* = 30), mucosal response (*n* = 40) or nonresponse (*n* = 7). B, Curve constructed from a probabilistic model determining specificity and sensitivity of a trough level >2.3 to predict mucosal healing in Crohn’s disease patients on maintenance ustekinumab therapy with an area under the curve of 0.95. *P < .001.

Studies of anti-TNF biologic drugs have identified that target serum trough levels associated with mucosal healing differ based on underlying patient phenotype such as UC vs CD^24-26^ or perianal vs luminal disease.^27,28^ Patients with CD were chosen as a distinct population to observe in this study, as there was an insufficient number of patients with confirmed UC/IBDU (*n* = 5) to power this analysis; and these were excluded from this study. Patients with mucosal healing (*n* = 30) had significantly higher mean ustekinumab levels (5.7 µg/mL, SD 6.4) compared with those with no response (1.1 µg/mL, SD 0.52; *n* = 7, *P* < .0001, Figure 1). Furthermore, higher mean trough ustekinumab levels (5.1 µg/mL, SD 6.1) were noted in patients with any mucosal response (*n* = 40) compared with nonresponse (1.1 µg/mL, SD 0.52; *n* = 7, *P* < .0001). We did not identify a trend towards higher mean serum ustekinumab levels in patients with perianal disease who achieved mucosal healing compared with patients with exclusively luminal disease, although the sample size may limit comparisons in this subpopulation.

Our probabilistic model constructed after logistic regression identified that serum ustekinumab trough levels greater than 2.3 µg/mL were 10.7 times more likely to be associated with MH (100% sensitivity; 95% CI, 71.5% to 100%; 90.6% specificity) and were 10.5 times more likely to achieve MR (100% sensitivity; 95% CI, 71.5% to 100%; 90.5% specificity) compared with nonresponse.

## Discussion

Studies indicate that 10% to 30% of patients with IBD have no initial response to anti-TNF therapy, and over 50% of those who do will lose response over time (secondary loss of response).^26^ Previously, research has indicated that serum concentration of anti-TNF biologic therapies is dose proportionate; this suggest an association between serum anti-TNF concentration level or neutralizing antibodies and objective endoscopic mucosal response.^29,30^ There are numerous studies reporting target trough levels of infliximab,^31^ adalimumab,^32^ and golimumab^22^ to achieve MH and some data investigating relationship between trough levels and outcomes with vedolizumab, an anti-integrin therapy.^33^ In contrast, there are few data supporting a correlation between serum ustekinumab levels and MH. The STARDUST randomized control trial^34^ is studying standard of care compared with treat-to-target ustekinumab therapy and has reported preliminary data at 1-year showing superiority of treat-to-target approaching achieving endoscopic response. The aim of this study was to determine whether dose escalation and higher ustekinumab levels may benefit patients with Crohn’s who have not achieved mucosal healing. In our observational cohort of patients with CD on maintenance ustekinumab, 63.8% of patients (*n* = 30 of 47) had achieved MH at time of level assay, and 85.1% (*n* = 40 of 47) had achieved at least MR. Seven patients in total exhibited nonresponse to ustekinumab dosing at the time of analysis. Our patient cohort was largely biologic-experienced, and a high proportion of patients with CD exhibited advanced luminal disease with stricturing or penetrating disease behaviour.

Previously published data has demonstrated that week-6 ustekinumab serum trough levels are associated with a change in surrogate biochemical markers of disease activity.^35^ A recent single-center prospective study^36^ in France involving 49 patients with CD on maintenance ustekinumab reported a mean serum ustekinumab serum trough concentration of 1.88 ± 1.40 µg/mL. There was no statistically significant difference reported in ustekinumab serum trough levels in patients with or without clinical, radiological, or endoscopic response to ustekinumab (*P* > .11). In contrast, another observational study^37^ of 62 nonresponders to anti-TNF therapy who then received subcutaneous ustekinumab induction reported that ustekinumab serum trough levels of 4.5 µg/mL at 26 weeks or later were associated with reduction in biomarkers and endoscopic response (67% sensitivity, 70% specificity; AUC, 0.67). In our study cohort, we report that an ustekinumab serum trough level greater than 2.3 µg/mL in patients with CD is associated with mucosal healing (SES-CD 0-5 or FC ≤150 µg/L), with sensitivity of 100% and specificity of 92.5% and a likelihood ratio of 13.3 (Figure 1B). Overall, mean serum ustekinumab trough levels in patients with mucosal healing or response compared with nonresponse were higher (*P* < .0001, Figure 1A).

There were no antidrug antibodies detected in the cohort, supporting speculation that the likelihood of developing anti-ustekinumab antibodies is less overall when compared with the traditionally observed rate of auto-antibody formation among patients who are maintained on TNF-α inhibitors.^38^ These data point to some potential benefit in using serum trough levels of ustekinumab to determine true nonresponse rather than insufficient dosing in patients who have not responded to ustekinumab. A further analysis of target levels or relationship between serum levels and MH in UC is also needed. However, we could not power this statistical analysis largely due to wider use of anti-integrin therapy and small molecule therapy as second- and third-line treatment options in this group at our centers. This reflects physician preference in some cases but also the financial restrictions around ustekinumab prescribing regionally. All decisions around ustekinumab dosing were at the discretion of the treating physician. A significant proportion of patients included in this study received higher than standard dosing. The most common reasons for change of dose frequency included patients who had achieved a clinical response but not remission, persistent endoscopic disease activity with improvement from baseline, and patients with aggressive perianal or penetrating disease who had previously required dose escalation of other biologic agents. The latter group, which was a minority, often received higher doses or shortened frequency from induction.

Moreover, only 45.3% of patients in the UNITI-1 study^18^ had a history of 1 anti-TNF treatment failure in comparison with 89.4% of our cohort. Induction strategies have comparable clinical response rates whether administered intravenously or subcutaneously.^39^ The UNITI 1, 2, and IM-UNITI studies^18^ and current approvals have led to current standard induction dosing for ustekinumab (6 mg/kg ustekinumab intravenously followed by 90 mg subcutaneously every 8 weeks). Pharmacokinetic analysis of ustekinumab in CD shows an association between higher serum ustekinumab concentrations and normalization of inflammatory biomarkers that are unaffected by prior biologic or immunomodulator use.^40^ A previous observational cohort study demonstrated that dose escalation to ustekinumab 4-weekly achieved clinical response in 61.1% of patients,^41^ and this influenced treatment strategies in our study where patients who required dose escalation were escalated to 6 (*n* = 4) and 4 (*n* = 12) weekly regimens (Table 2). A further 9 patients in the 4 weekly cohort progressed to a maximum dose of 180 mg, with a mucosal healing rate of 66.6% (*n* = 6) with at least mucosal response seen in 100% (*n* = 9) of this subgroup. Despite patients on escalated doses of 180 mg of ustekinumab 4-weekly and 8-weekly having significantly higher ustekinumab serum trough concentrations recorded, there was no statistically significant correlation between all higher ustekinumab doses including 90 mg 4-weekly and high trough levels, despite increased rates of mucosal healing. This may point to higher nonimmune-mediated drug clearance associated with greater inflammatory burden of patients for whom physicians may elect to use higher doses in response to disease therapy.^42^

**Table 2.** Maintenance subcutaneous ustekinumab doses prescribed to study subjects.

Ustekinumab dose 90mg	Ustekinumab dose 180mg
12 weekly 1.9% (*n* = 1)	8 weekly 1.9% (*n* = 1)
8 weekly 64.2% (*n* = 34)	4 weekly 17% (*n* = 9)
6 weekly 7.5% (*n* = 4)	
4 weekly 7.5% (*n* = 4)	

There were no adverse events (AEs) or side effects recorded for patients with high trough levels or who were maintained on a higher dose of ustekinumab or shorter frequency of drug administration. This finding is consistent with data published on higher serum trough levels in other biologic classes.^43^ Interestingly, we did not see any additional benefit to the use of combination therapy with immunomodulators or correlation between immunomodulator use and higher serum ustekinumab levels. This may relate to the lack of antibodies noted on the drug assay or point to differing pharmacokinetics between biologic classes and supports previously published data showing minimal, if any, benefit to combination therapy with ustekinumab.^44^ Our data on serum trough ustekinumab levels associated with mucosal healing or response in a predominantly treatment-experienced patient cohort may support the use of dose escalation or reduction in treatment interval in order to achieve mucosal healing in patients. This is particularly noteworthy for jurisdictions such as Ireland where reimbursement is limited to 90 mg doses every 8 weeks in many regions. Cost burdens associated with Crohn’s disease are influenced by biologic status due to higher frequency of admissions and outpatient visits.^45^ Despite this, there are hidden and overt financial barriers to biologic use and dose escalation of biologics for patients with Crohn’s disease.

This study contributes to the literature on the potential role or utility of serum ustekinumab levels within the overall therapeutic drug monitoring landscape informing bespoke treatment strategies of biologics for patients. These data identifying an association between serum ustekinumab levels and mucosal healing, particularly in CD, may support the use of proactive therapeutic drug monitoring to determine whether optimal dose regimes have been prescribed to increase likelihood of mucosal response.

Limitations of this study include the study’s retrospective design, the low number of biologic-naïve patients, the impact on timing to drug monitoring (largely due to timing of introduction of drug assay to our institutions), and the variable dosing regimens utilized, reflecting physician preference in this observational study. Strengths include its status as a dual-center study and the complex nature of disease detailed in this treatment-experienced cohort. This dual-center study associates higher ustekinumab serum trough levels with mucosal healing and mucosal response in patients with CD who are predominantly experienced with anti-TNF. Further prospective studies are required to correlate target maintenance trough levels and the optimal time to dose escalate to improve patient outcomes, as well as the potential impact of dual biologic therapy.
